# The dynamic association between body mass index and cognition from midlife through late-life, and the effect of sex and genetic influences

**DOI:** 10.1038/s41598-021-86667-4

**Published:** 2021-03-30

**Authors:** Ida K. Karlsson, Margaret Gatz, Thalida Em Arpawong, Anna K. Dahl Aslan, Chandra A. Reynolds

**Affiliations:** 1 Institute of Gerontology and Aging Research Network-Jönköping (ARN-J), School of Health and Welfare, Jönköping University, Jönköping, Sweden; 2 Department of Medical Epidemiology and Biostatistics, Karolinska Institutet, Stockholm, Sweden; 3 Center for Economic and Social Research, University of Southern California, Los Angeles, CA USA; 4 Leonard Davis School of Gerontology, University of Southern California, Los Angeles, CA USA; 5 Department of Health Sciences, School of Health Sciences, University of Skövde, Skövde, Sweden; 6 Department of Psychology, University of California, Riverside, USA

**Keywords:** Psychology, Risk factors, Epidemiology

## Abstract

Body mass index (BMI) is associated with cognitive abilities, but the nature of the relationship remains largely unexplored. We aimed to investigate the bidirectional relationship from midlife through late-life, while considering sex differences and genetic predisposition to higher BMI. We used data from 23,892 individuals of European ancestry from the Health and Retirement Study, with longitudinal data on BMI and three established cognitive indices: mental status, episodic memory, and their sum, called total cognition. To investigate the dynamic relationship between BMI and cognitive abilities, we applied dual change score models of change from age 50 through 89, with a breakpoint at age 65 or 70. Models were further stratified by sex and genetic predisposition to higher BMI using tertiles of a polygenic score for BMI (PGS_BMI_). We demonstrated bidirectional effects between BMI and all three cognitive indices, with higher BMI contributing to steeper decline in cognitive abilities in both midlife and late-life, and higher cognitive abilities contributing to less decline in BMI in late-life. The effects of BMI on change in cognitive abilities were more evident in men compared to women, and among those in the lowest tertile of the PGS_BMI_ compared to those in the highest tertile, while the effects of cognition on BMI were similar across groups. In conclusion, these findings highlight a reciprocal relationship between BMI and cognitive abilities, indicating that the negative effects of a higher BMI persist from midlife through late-life, and that weight-loss in late-life may be driven by cognitive decline.

## Introduction

We aimed to investigate the direction of the relationship between body mass index (BMI) and cognition from midlife through late-life, and whether these associations vary with sex or genetic predisposition to high BMI. While overweight and obesity in midlife have well-demonstrated associations with lower cognitive abilities, there is also evidence indicating that lower cognition is associated with an unhealthy lifestyle which may lead to higher BMI^1,2^. From both a biological and a public health perspective it is important to understand the chains of processes that lead to change in cognitive abilities as well as to weight changes, so that interventions can be directed to the right level. Currently, some studies support that change in BMI influences change in cognitive abilities, and the vice versa, but to the best of our knowledge only one previous study has explored the bidirectionality between BMI and cognitive abilities in adulthood. This study, by Hartanto and colleagues, demonstrated bidirectionality in the linear association between overweight and cognitive abilities between two assessments over 9 years apart in individuals aged 33–84 at baseline^1^. However, to understand the dynamic relationship between BMI and cognitive abilities, i.e. how change in BMI is associated with change cognitive abilities and vice versa, data with several measurement points are necessary. By taking advantage of the repeated measures in the Health and Retirement Study (HRS) we will expand current knowledge within the field by exploring the dynamic association between BMI and cognitive abilities.

We also need to be aware of age because substantial changes occur in both BMI and cognitive abilities across the second half of the lifespan. Some degree of cognitive decline may be a natural part of the aging process, but there is great variability. Some individuals maintain a stable cognitive ability throughout late-life whereas others experience decline to such a degree that it interferes with daily functioning and is hence considered dementia^3^. BMI tends to increase across adulthood through age 65, after which it levels out to then start to decline around age 80^4^. In contrast to midlife, where high BMI is considered to be a risk factor, a high BMI after age 65 is generally associated with better health outcomes, including higher cognitive abilities^5^.

As sex differences are present in both BMI^4^ and cognitive abilities^6^ across adulthood, associations between the two phenotypes must be considered by sex. How BMI reflects body fat may differ for men and women. In turn, body fat distribution differs between men and women, which is of importance as where, and what type of fat is stored affects the association between overweight and negative health outcomes^7^. In line with this point, there is evidence, albeit inconsistent, that overweight may contribute to negative health outcomes differently in men and women^8,9^. It is also plausible that cognitive ability influences body weight differently in men and women^10^.

Both overweight and cognition are complex phenotypes influenced by genetic as well as environmental factors^11,12^. According to twin studies, between 45 and 85% of the variance in body mass index (BMI)^11^ and around 50% of the variance in cognitive abilities^12^ is explained by genetic factors. Marioni and colleagues^13^ demonstrated a significant genetic overlap between BMI and cognition, such that genetic variants related to higher cognitive ability were also correlated with a lower BMI. It is thus plausible that the two phenotypes are influenced by common biological mechanisms, partly explaining their association.

Taken together, age, sex, and genetic factors complicate the interpretation of associations between BMI and cognitive abilities. Thus, we here aimed to carefully examine the dynamic relationship between BMI and cognitive abilities by studying age-specific and non-linear effects from midlife through late-life, while also considering differences based on sex and genetic predisposition to a higher BMI. Specifically, we aimed to (1) explore the direction of the relationship (whether BMI predicts change in cognitive ability or if cognitive ability predicts change in BMI, or if the relationship is of a reciprocal nature), considering up to 11 assessments across 21 years and whether the relationship differs with age, (2) examine whether the associations differ between men and women, and (3) examine whether the associations differ by genetic predisposition to low, medium, or high BMI.

## Methods

### Study population

This study used longitudinal data from the Health and Retirement Study (HRS)^14^, a nationally representative sample of adults aged 50+ in the US and their spouses, and an open-access data source with funding from the National Institutes of Health^14^. The HRS and the Asset and Health Dynamics (AHEAD) Study began in 1992 and 1993, respectively, and merged in 1998. Both studies use a panel design in which the same individuals are interviewed every 2 years, with new respondents added every 6 years as replenishment samples to account for aging and attrition. We used data from the HRS cohort from 1996 onwards and from the AHEAD cohort from 1993 onwards, through wave 12 in 2014. Data from the first two waves of the HRS (1992 and 1994) were excluded from the current analyses because cognitive assessments were administered in a different format. We also excluded data assessed prior to age 50 for the respondent and where individuals required a proxy to complete their interview. We encountered problems with model convergence when including individuals who specified “African” or “other” descent, as only 1998 had at least three measurement points (the minimum required to contribute to change parameters) and only 953 of these had genetic information available. Thus, the current study includes data from a sample of 28,148 individuals with European ancestry, who had up to 11 waves per participant, with identical cognitive measures, and covariates available.

Respondent information, including cognitive information, was obtained from the RAND HRS Longitudinal File 2014 (V3)^15,16^, that combines the HRS core data files for each wave, which was merged with the BMI polygenic score (PGS) and BMI data retrieved and cleaned from the RAND HRS Fat Files (1992–2014)^15,17^.

The HRS data collections are conducted in accordance with the Declaration of Helsinki. All participants provided informed consent, and the HRS is approved by the Institutional Review Board at the University of Michigan.

### BMI measurements

BMI is calculated as weight (kilograms), divided by height (meters) squared, kg/m^2^. Height is self-reported at baseline for each participant and carried forward for each subsequent wave, and weight is self-reported at each wave. Hence, BMI is available on a biennial basis. Use of self-reported height and weight to calculate BMI, specifically in longitudinal aging studies, has been validated against in-person measures previously^18^. The BMI data were cleaned prior to analyses, the process described in detail elsewhere^19^, removing outliers (1515 individuals, or 4% of the total HRS sample) including those that had values considered out-of-range (e.g., height below 1.47 or above 2.10 m, BMI below 15 or above 55) and unreasonably large changes between waves. BMI at baseline was categorized into normal weight (18.5–24.9), overweight (25–29.9), and obesity (30 and above).

### Cognitive measurements

The HRS protocols include a series of cognitive tests, administered either in person or over the telephone, based on a modified version^20^ of the Telephone Interview for Cognitive Status (TICS)^21^. A prior randomization study has shown that there are no significant effects on cognitive performance based on mode of interview (telephone or in person)^20^. From the cognitive tests, three summary indices have been computed by the HRS^20^. To minimize issues with cognitive information not missing at random, HRS imputed missing values on the summary indices across waves for individuals with incomplete cognitive test information based information about demographics, health, economic circumstances, prior cognitive scores and current scores on non-missing items. If the participant was unable to complete the interview, information from proxy interviews was not used for imputation^22^. A measure of *episodic memory* was calculated as a total recall score from summing the total number of words recalled immediately after being read a list of 10 nouns, and after a short delay (score range 0–20). The episodic memory assessments were completed in HRS at each wave. A *mental status* index was calculated by summing scores from a serial 7 s test of working memory, counting backwards to assess attention and processing speed, and naming tasks (today’s date, objects, and the President and Vice President) to assess language and orientation (score range 0–15). This index is generally stable through midlife and early late-life. Therefore, the mental status assessments in HRS were administered at baseline to everyone, then again at age 65 and every wave thereafter. A total cognition index was calculated by summing the episodic memory and mental status indices (score range 0–35).

Based on the dementia definition developed by Langa and Weir^23^ (using the cognitive tests and validated cut-points^24^), we censored cognitive test scores beginning at age of dementia onset. For the present study, all scores were converted to T-scores with mean 50 and standard deviation 10, scaled to a reference sample of individuals age 50 or older (mean = 66.5, standard deviation (SD) = 10.0) participating in the third wave of HRS (as the sample size in earlier waves was limited). Cognitive ability at baseline was also categorized into a level of below 50, or 50 and above.

### Polygenic scores (PGSs)

Genotyping was performed by the NIH Center for Inherited Disease Research (CIDR; Johns Hopkins University, Baltimore, MD) using the Illumina Human Omni2.5-Quad BeadChip (Illumina, San Diego, CA), with coverage of nearly 2.5 million single nucleotide polymorphisms (SNPs). All quality control checks were implemented by the HRS. From these data, the HRS calculates and distributes PGSs for several phenotypes, including BMI^25^. The PGS_BMI_ was generated based on a GWAS meta-analysis for BMI^26^ by the Genetic Investigation of ANthropometric Traits (GIANT) consortium on 339,224 individuals. The HRS sample was also part of the BMI GWAS, and it should be noted that the PGS_BMI_ was generated without removing the sample overlap. While this could lead to over inflation of its predictive ability^27^, the PGS is used here only to categorize individuals by genetic predisposition to higher BMI. The PGS_BMI_ is available for 12,090 participants of European ancestry from the study sample.

To minimize issues related to population substructure, the HRS conducts principal components analysis (PCA) to identify population group outliers and provide principal components as covariates for genetic analyses^28^. Thus, the PGS_BMI_ was adjusted for genetic ancestry by regressing out the first five principal components, and then standardized to mean = 0 and SD = 1. Individuals were then categorized by tertiles of the PGS_BMI_, into those with genetic predisposition to low, medium, and high BMI.

### Statistical analyses

The dynamic association between BMI and cognitive abilities was modelled in dual change score models (DCSM) in a strategy laid out by McArdle and colleagues^29,30^ using Mplus^31^. DCSMs are an extension of latent growth curve models, but developed to study dynamics. The bivariate DCSM examines the direction of associations by considering the timing of measurements and change, testing whether within-person change in one variable temporally precedes change in the other. With this model we fitted an implied trajectory of each construct separately (i.e., BMI and cognitive functioning) and included coupling parameters to test if changes in the constructs are associated within time and dynamically linked over time. The static portion of the DCSM estimates an intercept and slope that considers the maturational changes that play out across time. The implied trajectories are associated through these static factors as well. The coupling effects then consider change above and beyond the static components. The temporal nature of the data provides leverage that, after accounting for the static components, some directional temporal dynamics can be supported above others, or, that there are bidirectional dynamics that play out across time. If there were no causal connections and it was simply a matter of correlated slopes, then only the static portion would be salient. Coupling effects are thus consistent with, but not proof of, a causal relationship between the processes.

For modelling purposes, the data were split into 2-year age intervals by age at measurement of BMI and cognitive ability, ranging from 50–51.99 through 88–89.99 (here forth referred to as age 50–51 through 88–89). Should an individual have two measurements within the same age category, the first measurement occasion was used. The number of individuals in each age category is shown in Table S1, along with the total number of individuals with any number of measurement occasions and with at least three measurements. While all individuals are included in the DCSM and contribute to the mean level of respective variable, a minimum of three measurement occasions are required of individuals to contribute to the change parameters. In all models described below, intercept level and the constant linear slope for BMI and cognitive abilities were adjusted for sex and education. Age was adjusted for by using age in 2-year bins as the underlying timescale, and estimates of change thus represent units of change over 2 years. Further, the clustering of individuals in households was accounted for through robust standard errors.

**Figure 1:**
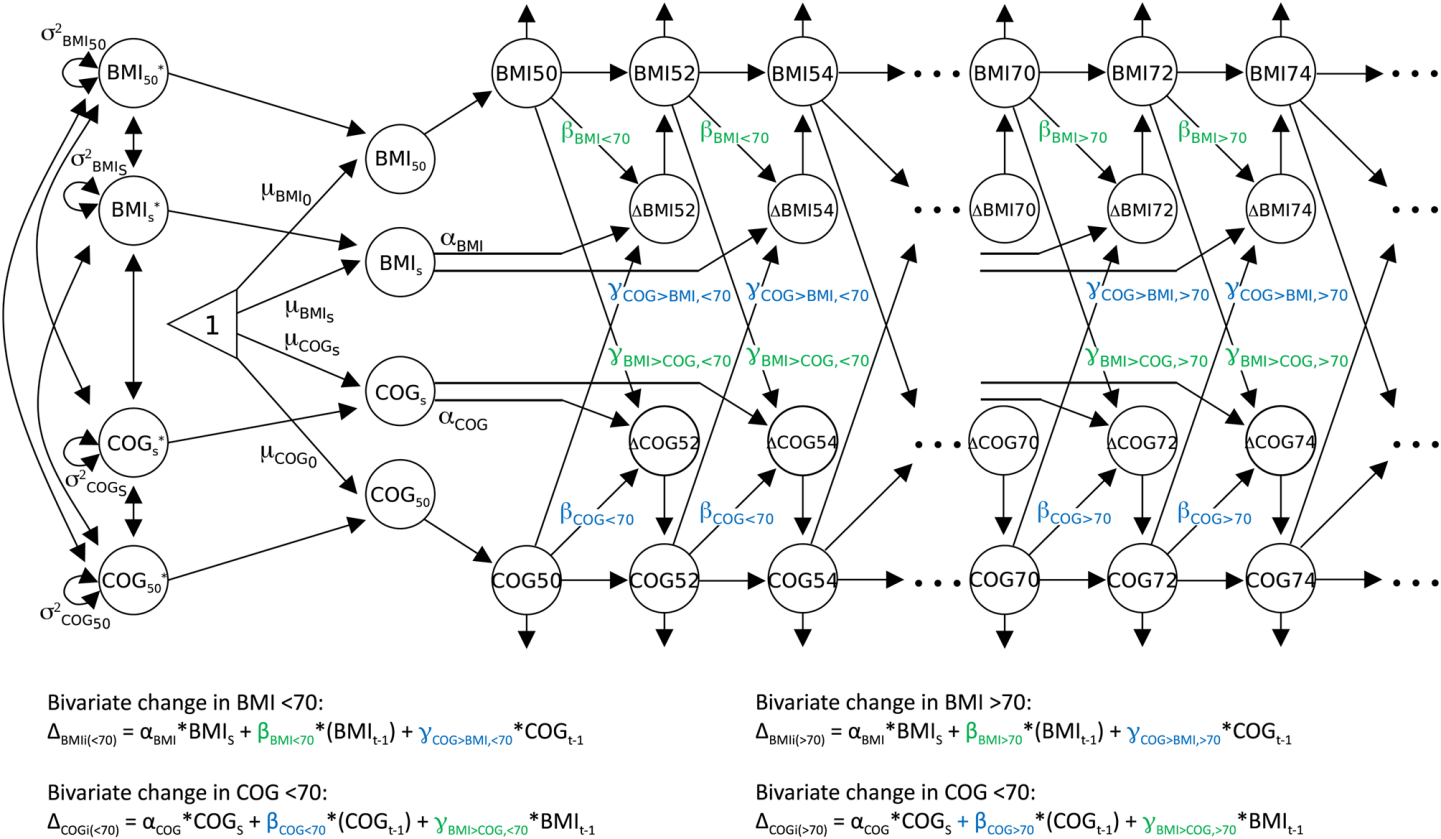
Path diagram of the bivariate dual change score model with two slopes, allowing for different rates of change before and after age 70. Body mass index (BMI) and cognitive abilities (COG) in each age category (BMI50, BMI52…; COG50, COG52…) are modeled. From the left, BMI_50_, BMI_S_, COG_50_, and COG_S_ represent intercept level (at age 50) and slope of BMI and cognitive ability (asterisks represent their standardized scores), together with their estimated mean levels ($\mu_{{{\text{BMI}}_{50} }}$, $\mu_{{{\text{BMI}}_{{\text{S}}} }}$, $\mu_{{{\text{COG}}_{{{5}0}} }}$, $\mu_{{{\text{COG}}_{{\text{S}}} }}$) and variances ($\sigma^{2}_{{{\text{BMI}}_{50} }}$, $\sigma^{2}_{{{\text{BMI}}_{{\text{S}}} }}$, $\sigma^{{2}}_{{{\text{COG}}_{{{5}0}} }}$, $\sigma^{{2}}_{{{\text{COG}}_{{\text{S}}} }}$). α_BMI_ and α_COG_ represent the constant linear change related to the slopes, and β_BMI_ and β_COG_ represent the proportional non-linear change. γ_BMI>COG_ represent the coupling effect of BMI on cognition, and γ_COG>BMI_ that of cognition on BMI. Univariate change in BMI at age = i, before age 70, is determined by the sum of the constant change in relation to the slope (α_BMI_ * BMI_S_) and the proportional change in relation to BMI level at the preceding occasion (β_BMI<70_ * BMI_t−1_). For bivariate change in BMI, considering the effect of cognitive ability, the additional coupling effect in relation to cognitive ability at the preceding occasion is added to the formula (γ_COG>BMI, <70_ * COG_t−1_). Univariate and bivariate change in BMI after age 70 and in cognitive abilities before and after age 70 is determined by the same formulas, using the corresponding parameters.

A path diagram of the DCSM model is depicted in Fig. 1. Univariate DCSMs were first applied separately to BMI and the cognitive abilities to examine the linear and non-linear changes in the respective phenotype across age. The univariate DCSMs for BMI and cognitive abilities are visualized in the top and bottom part of Fig. 1, respectively. The univariate DCSM for BMI is built of latent difference scores (BMI_t_) with change from one age to the next generating a growth curve (Δ_BMIt_), which can be written as:

$$\Delta_{BMIt} = \alpha_{BMI} \times BMI_{s} + \beta_{BMI} \times BMI_{t - 1}$$

i.e. the constant, linear change component which consists of the $\alpha_{BMI}$ parameter multiplied by the linear slope factor $BMI_{s}$, plus the proportional change $\beta_{BMI}$ parameter multiplied with BMI level at the previous measurement. The α parameter is normally set to 1, and the β parameter is thus a measure of the non-linear change. The linear and proportional change features are assumed to persist over time, and while the linear change component is allowed to vary over persons ($\sigma^{2}_{{{\text{BMI}}_{{\text{S}}} }}$), the proportional change parameter $\beta_{BMI}$ is fixed across persons. In addition to change in BMI, the model estimates the mean intercept level ($\mu_{{{\text{BMI}}_{50} }}$), mean slope ($\mu_{{{\text{BMI}}_{{\text{S}}} }}$), the variance of the intercept ($\sigma^{2}_{{{\text{BMI}}_{50} }}$) and slope ($\sigma^{2}_{{{\text{BMI}}_{{\text{S}}} }}$), the residual variance (σ^2^_BMI_), and the correlation between the intercept and slope. The univariate DCSM for cognition is built of corresponding parameters.

To examine differences in change across age, univariate models with breakpoints after age 64–65, 68–69, and 74–75, approximately corresponding to breakpoints 65, 70 and 75 years, were tested. This allows for different proportional effects ($\beta_{BMI}$, $\beta_{COG}$) before and after the breakpoint. It should be noted that the constant, linear slope does not differ before and after the break-point. The best fitting model with respect to age breakpoints (based on the Akaike information criteria (AIC)) was compared to the null-model without a breakpoint for age, and selected for further models if it had significantly better fit. This and following tests of significance of change in model fit were tested by the log-likelihood difference test with the MLR correction for scaling factors (as the MLR log likelihood does not follow a χ^2^ distribution), described by Satorra and Bentler^32^. A technical complication with the approach is that the computation can sometimes produce negative χ^2^ test statistics. When arising, this issue was dealt with by instead comparing to a more restricted model, marking such instances in the tables of results.

Secondly, bivariate DCSMs were applied to study the dynamic association between BMI and cognitive abilities. In addition to the univariate components described above, the bivariate DCSM estimates the covariance between intercepts and slopes for BMI and cognition, and two coupling parameters (γ_COG>BMI_ and γ_BMI>COG_). While the covariances measure static associations between the two constructs, the γ_COG>BMI_ parameter measure dynamic associations by estimating how level of cognitive ability at t = i−1 is associated with change in BMI level from t = i−1 to t = i (shown in Fig. 1 as arrows from COG_t−1_ to Δ_BMIt_).

Change in BMI at age = t as a function of prior level of cognitive ability can thus be written as:

$$\Delta_{BMIt} = \alpha_{BMI} \times BMI_{s} + \beta_{BMI} \times BMI_{t - 1} + \gamma_{COG \to BMI} \times COG_{t - 1}$$

As with the α and β parameters, the γ parameters are assumed to persist over time (but allowed to differ before and after a breakpoint). The bivariate DCSM for cognition is built of corresponding parameters.

The temporal order of changes, and thus direction of effects, can be tested by restricting the coupling parameters and testing whether doing so significantly reduced the model fit. First, a full-coupling (bidirectional) model was tested, with both coupling parameters (γ_BMI>COG_ and γ_COG>BMI_) included. Secondly, a no-coupling model was tested, with none of the coupling parameters included (i.e. only the static proportion of the DCSM). We then compared model fit (as described above) to test for the presence of any dynamic association between BMI and cognitive ability. When evidence of a dynamic association was present, a third and fourth model were tested, including only one of the coupling parameters and thus testing for a unidirectional effect.

Lastly, we tested for sex differences and the influence of genetic predisposition to higher BMI, by studying group differences across (1) sex, and (2) tertiles of the PGS_BMI_, respectively. Here, we first tested for group-specific effects in the univariate models, by comparing a model where all parameters were free to vary across groups to models where the following parameters were constrained to be group-invariant in a stepwise manner: (1) residual variances, (2) variances and covariances, (3) the proportional change parameter, and (4) the mean intercept and slope. To test the significance of group effects on the parameters, we compared the fit of the more constrained group-invariant model to that of the previous model with parameter/parameters allowed to vary freely across groups.

Next, differences in the bivariate associations by (1) sex, and (2) tertiles of the PGS_BMI_ were tested in a similar manner. A model where all univariate and bivariate parameters were free to vary across groups was compared to models where the bivariate parameters were constrained to be group-invariant in the following step-wise manner: (1) both coupling-parameters (bidirectional), (2) the residual covariance, and (3) cross-trait covariance between intercepts and slopes (i.e. all bivariate parameters). Where the bidirectional coupling-parameters showed significant group differences we also tested for unidirectional group differences. Across all models, the univariate parameters were free to vary across groups to avoid inflation of group effects on the univariate parameters.

Results from DCSM were extracted with the MplusAutomation package^33^ and trajectories plotted with the ggplot2 package^34^ in in R 4.0.3^35^.

## Results

### Study population

After removing individuals with dementia at baseline (n = 932), no cognitive data on any of the three indices (n = 1971), no BMI data (n = 869), or neither cognitive nor BMI data between age 50 and 89 (n = 520), 23,892 individuals remained in the final analysis sample. The analyses include individuals who subsequently became demented, but not their data from when they met the Langa–Weir cutoff for dementia^23^ and onwards. Mean age at first and last wave of participation for the same individuals was 62.0 years (range 50.0–90.0, SD = 9.9) and 72.5 years (range 50.0–90.0, SD = 10.8), respectively, with a mean of 6.1 measurement occasions (range 1–11, SD = 3.3). Mean BMI at the baseline age was 27.2 (range 15.1–54.9, SD = 5.2). The sample consisted of 13,309 women (55.7%) and 10.583 men (44.3%), and 13,294 individuals (55.6%) had high school education or less, while 10,598 (44.6%) had some college education or more.

### Findings from DCSM analyses

Given the volume of analyses and output, only the main findings are presented within the article, while detailed results are presented in the supplementary tables and figures with a brief description here.

#### Univariate trajectories of BMI and cognitive abilities

A model with different proportional effects before and after a breakpoint had significantly better fit across all models (p < 0.001) than a model with no breakpoint, indicating acceleration of change or a switch from gain to loss after the designated breakpoint age. A breakpoint at age 70 showed the best fit for BMI and mental status, while a breakpoint at age 65 best fit episodic memory and total cognition (Table S2).

The univariate trajectory parameters for BMI and cognitive abilities are presented in Table 1a–c and trajectories visualized in Figure S1a–d. The full model estimates are presented in Table S3a–d. At age 50, mean BMI was 27.8; BMI level then increased until the age 70 breakpoint, after which it started to decline. Mean mental status at age 50 was 51.1; scores remained rather stable up to age 70, and then started to decline. Episodic memory and total cognition started at mean 49.9 and 50.1, respectively, decreased through age 65, followed by a steeper decrease after the breakpoint.

**Table 1:** Univariate and bivariate change in body mass index and cognitive abilities from age 50–89 in the Health and Retirement Study.

	Univariate		Bivariate	
	Estimate	SE	Estimate	SE
**(a) Mental status**				
BMI parameters				
Mean intercept level ($\mu_{{{\text{BMI}}_{50} }}$)	27.77*	0.07	27.69*	0.07
Mean slope ($\mu_{{{\text{BMI}}_{{\text{S}}} }}$)	− 0.36*	0.11	− 18.04*	1.24
Proportional effect < 70 (β_BMI<70_)	0.02*	0.00	− 0.45*	0.04
Proportional effect > 70 (β_BMI>70_)	0.00	0.00	− 0.45*	0.04
Cognition parameters				
Mean intercept level ($\mu_{{{\text{COG}}_{{{5}0}} }}$)	51.12*	0.13	50.43*	0.11
Mean slope ($\mu_{{{\text{COG}}_{{\text{S}}} }}$)	− 9.98*	0.72	− 6.25*	1.08
Proportional effect < 70 (β_COG<70_)	0.20*	0.01	0.36*	0.04
Proportional effect > 70 (β_COG>70_)	0.19*	0.01	0.36*	0.04
Bivariate parameters				
Coupling effect BMI on cognition < 70 (γ_BMI>COG, <70_)	–	–	− 0.43*	0.03
Coupling effect BMI on cognition > 70 (γ_BMI>COG, >70_)	–	–	− 0.43*	0.03
Coupling effect cognition on BMI < 70 (γ_COG>BMI, <70_)	–	–	0.61*	0.04
Coupling effect cognition on BMI > 70 (γ_COG>BMI, >70_)	–	–	0.61*	0.04
**(b) Episodic memory**				
BMI parameters				
Mean intercept level ($\mu_{{{\text{BMI}}_{50} }}$)	27.77*	0.07	27.40*	0.08
Mean slope ($\mu_{{{\text{BMI}}_{{\text{S}}} }}$)	− 0.36*	0.11	− 3.67*	0.96
Proportional effect < 70 (β_BMI <70_)	0.02*	0.00	− 0.02	0.05
Proportional effect > 70 (β_BMI >70_)	0.00	0.00	− 0.06	0.04
Cognition parameters				
Mean intercept level ($\mu_{{{\text{COG}}_{{{5}0}} }}$)	49.88*	0.13	49.51*	0.50
Mean slope ($\mu_{{{\text{COG}}_{{\text{S}}} }}$)	− 3.86*	0.25	− 0.67	3.64
Proportional effect < 65 (β_COG <65_)	0.07*	0.01	0.09*	0.01
Proportional effect > 65 (β_COG >65_)	0.07*	0.01	0.07*	0.01
Bivariate parameters				
Coupling effect BMI on cognition < 65 (γ_BMI>COG, <65_)	–	–	− 0.15	0.11
Coupling effect BMI on cognition > 65 (γ_BMI>COG, >65_)	–	–	− 0.12	0.11
Coupling effect cognition on BMI < 70 (γ_COG>BMI, <70_)	–	–	0.09	0.05
Coupling effect cognition on BMI > 70 (γ_COG>BMI, >70_)	–	–	0.12*	0.05
**(c) Total cognition**				
BMI parameters				
Mean intercept level ($\mu_{{{\text{BMI}}_{50} }}$)	27.77*	0.07	27.49*	0.08
Mean slope ($\mu_{{{\text{BMI}}_{{\text{S}}} }}$)	− 0.36*	0.11	− 2.77*	0.32
Proportional effect < 70 (β_BMI <70_)	0.02*	0.00	0.00	0.01
Proportional effect > 70 (β_BMI >70_)	0.00	0.00	− 0.04*	0.01
Cognition parameters				
Mean intercept level ($\mu_{{{\text{COG}}_{{{5}0}} }}$)	50.09*	0.14	50.26*	0.14
Mean slope ($\mu_{{{\text{COG}}_{{\text{S}}} }}$)	− 6.23*	0.26	− 4.79*	0.81
Proportional effect < 65 (β_COG <65_)	0.12*	0.01	0.15*	0.01
Proportional effect > 65 (β_COG >65_)	0.12*	0.01	0.12*	0.01
Bivariate parameters				
Coupling effect BMI on cognition < 65 (γ_BMI>COG, <65_)	–	–	− 0.10*	0.03
Coupling effect BMI on cognition > 65 (γ_BMI>COG, >65_)	–	–	− 0.06*	0.03
Coupling effect cognition on BMI < 70 (γ_COG>BMI, <70_)	–	–	0.06*	0.01
Coupling effect cognition on BMI > 70 (γ_COG>BMI, >70_)	–	–	0.08*	0.01

*SE* standard deviation, *BMI* body mass index, *COG* cognition.

Parameter estimates and standard deviations from the univariate and bivariate dual change score models of BMI and cognitive abilities in 23,892 individuals of European ancestry from the Health and Retirement Study. The slope parameters represent the mean linear change over 2 years. Proportional and coupling effects represent additional change over 2 years in relation to within and across-trait levels, respectively, at the preceding time point. BMI parameters are in BMI units and cognition parameters in T-score units. Models are adjusted for sex and education. A breakpoint was included to allow for differences in the proportional change and coupling parameters (mean slope is constant across age) before and after age 70 for BMI and mental status, and at 65 for episodic memory and total cognition. *p < 0.05.

##### Sex-differences

For both BMI and the cognitive outcomes, significant sex differences were identified on all univariate parameters tested (mean intercept and slope, proportional change, variances and covariances, and residual variances; Table S4; p < 0.001 for all). Univariate trajectories by sex are shown in Figure S2a–d, with the full model estimates presented in Table S3a–d. Compared to men, women had lower BMI at age 50, a larger increase in BMI through age 70, followed by a steeper decline from age 70 through 89. Women had lower mean level mental status across the age range, with slightly (although statistically significant) less decline over time. For both episodic memory and total cognition, women had higher levels of performance than men at age 50, followed by a similar rate of change up to age 65, but a steeper decline than men after age 65.

##### Differences by genetic predisposition to higher BMI

Stratifying by tertiles of the PGS_BMI_ yielded significant group differences on all univariate parameters for BMI (Table S4; p < 0.001 for all). Individuals with a high PGS_BMI_ had higher mean BMI level at age 50, similar increase through age 70, followed by a steeper decline between age 70 and 89, compared to those in the lower categories (Figure S3a, full model estimates in Table S3a). The overall effect of the PGS_BMI_ on phenotypic BMI level thus decreased after age 70. Across all cognitive outcomes, significant group differences by PGS_BMI_ were identified for mean level and slope, but not for the other univariate parameters (Table S4). As seen in Figure S3b–d (full model estimates in Table S3b–d), those in the low PGS_BMI_ group had slightly higher cognitive level at age 50. Only small group differences in the slopes were observed, with the largest effect on visualized trajectories for mental status (Figure S3b–d).

#### Bivariate trajectories of BMI and cognitive abilities

**Figure 2:**
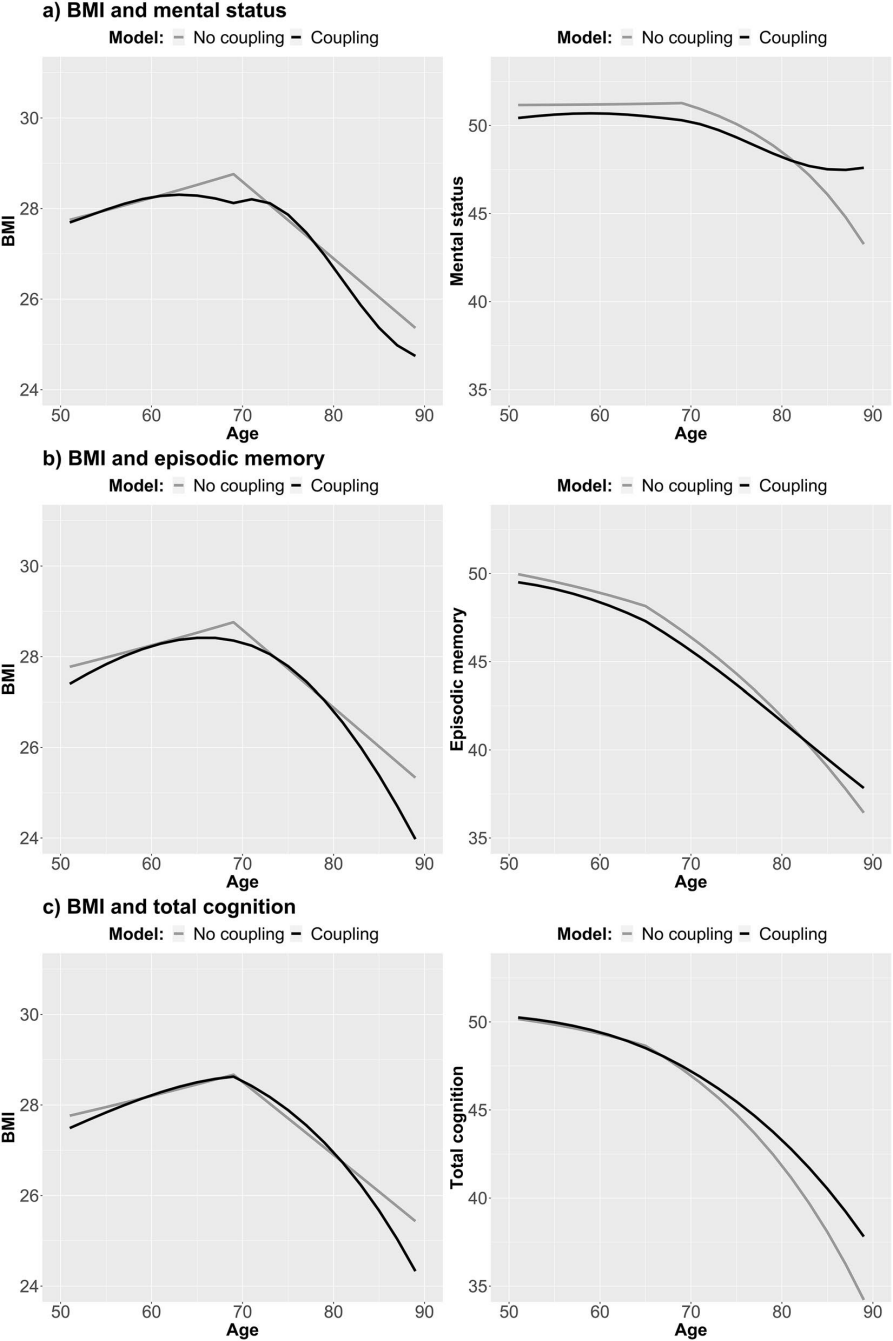
Longitudinal trajectories from bivariate dual change score models, showing change in BMI and cognitive abilities with and without the bivariate coupling-parameter. Trajectories from the full coupling model are shown in black, and those from the no-coupling model in grey. Models were adjusted for sex and education, and a breakpoint in the proportional change parameter added at age 70 for BMI and mental status, and at 65 for episodic memory and total cognition.

Longitudinal trajectories of change in BMI and respective cognitive outcome, with and without the bivariate coupling parameters considered, are visualized in Fig. 2. Here, the no-coupling trajectories correspond to those from the univariate models, while the full-coupling trajectories show change in BMI when the effect of cognitive ability is considered, and change in cognitive abilities when considering the effect of BMI. The bivariate trajectory estimates for BMI and cognitive abilities are presented in Table 1 alongside those from the univariate models, with the full model estimates presented in Table S6a–c.

The full coupling model showed significantly better fit compared to the no-coupling model across all cognitive outcomes, as well as to the unidirectional models (p < 0.01 for all; Table S5). Thus, the dynamic relationship between change in BMI and the three cognitive outcomes are of a bidirectional nature, with BMI driving change in cognitive abilities, while cognitive abilities also drive change in BMI. It is important to note that change in respective parameter is made up by the linear slope (BMI_S_ or COG_S_), proportional change (β_BMI_ or β_COG_), and coupling parameter (γ_COG>BMI_ or γ_BMI>COG_), and all must be considered together. The effect of coupling on the linear slope and proportional change parameters can be seen by comparing the parameters from the univariate and bivariate results in Table 1.

**Figure 3:**
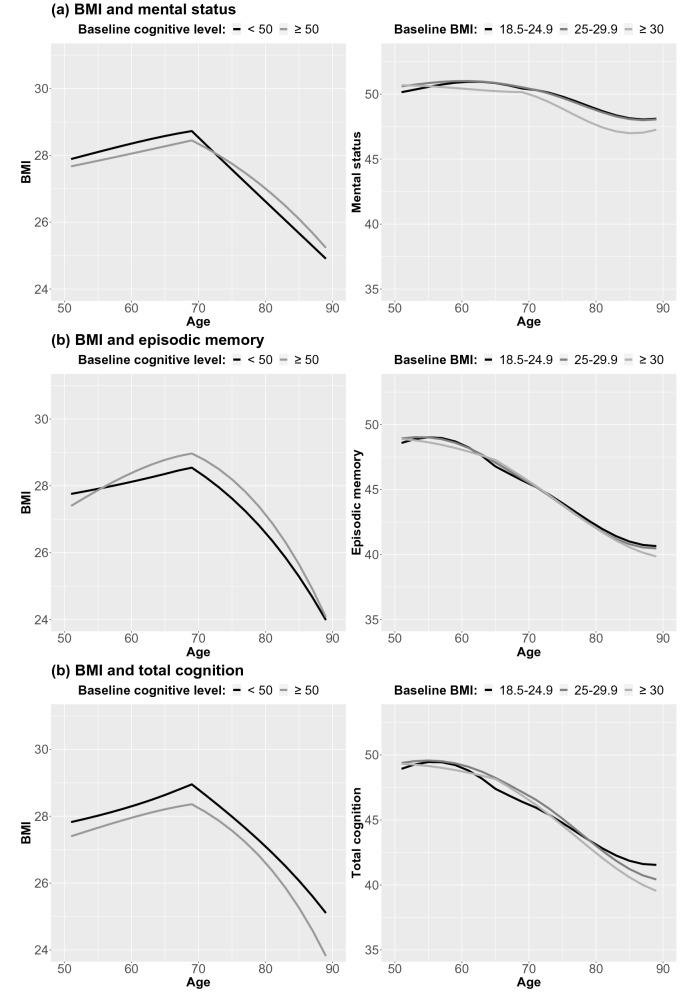
Longitudinal trajectories from bivariate dual change score models, showing change in BMI stratified by cognitive ability level at baseline, and change in cognitive abilities stratified by BMI level at baseline. Models were adjusted for sex and education, and a breakpoint in the proportional change and coupling parameters added at age 70 for BMI and mental status, and at 65 for episodic memory and total cognition.

Mental status showed the strongest association with change in BMI (Table 1a). When the effect of cognition was considered, the linear slope and proportional effects of BMI were markedly more negative, but this steeper decline was buffered by a positive coupling effect, where higher mental status predicted less decrease in BMI (γ_COG>BMI_ = 0.61 both before and after the age 70 breakpoint; Table 1a). The effect of BMI on mental status was smaller, but indicated that when the effect of BMI was considered, there was overall less decline in mental status, but that the decline was steeper with a higher BMI (γ_BMI>COG_ = − 0.43 both before and after the age 70 breakpoint; Table 1a). The same pattern can be seen in Fig. 3, which visualizes bivariate trajectories in BMI stratified by baseline level of cognitive abilities (left half of figure), and bivariate trajectories in cognitive abilities, stratified by baseline level of BMI (right half of figure).

Compared to those with mental status level below 50 at baseline, those with a baseline level of 50 or above had lower BMI level at baseline, comparable increase between age 50 and 70, but less decline from age 70 through 89. Stratifying trajectories of mental status by baseline level of BMI demonstrated a steeper decline already from age 50 among those with BMI of 30 or above compared to those in the lower categories.

The parameters for episodic memory and total cognition were in the same direction as for mental status, but substantially weaker. The coupling parameters (γ_COG>BMI_ and γ_BMI>COG_) were estimated to between 0.06 and 0.10, and − 0.06 and − 0.12, respectively (Table 1b,c).

##### Sex-differences

**Figure 4:**
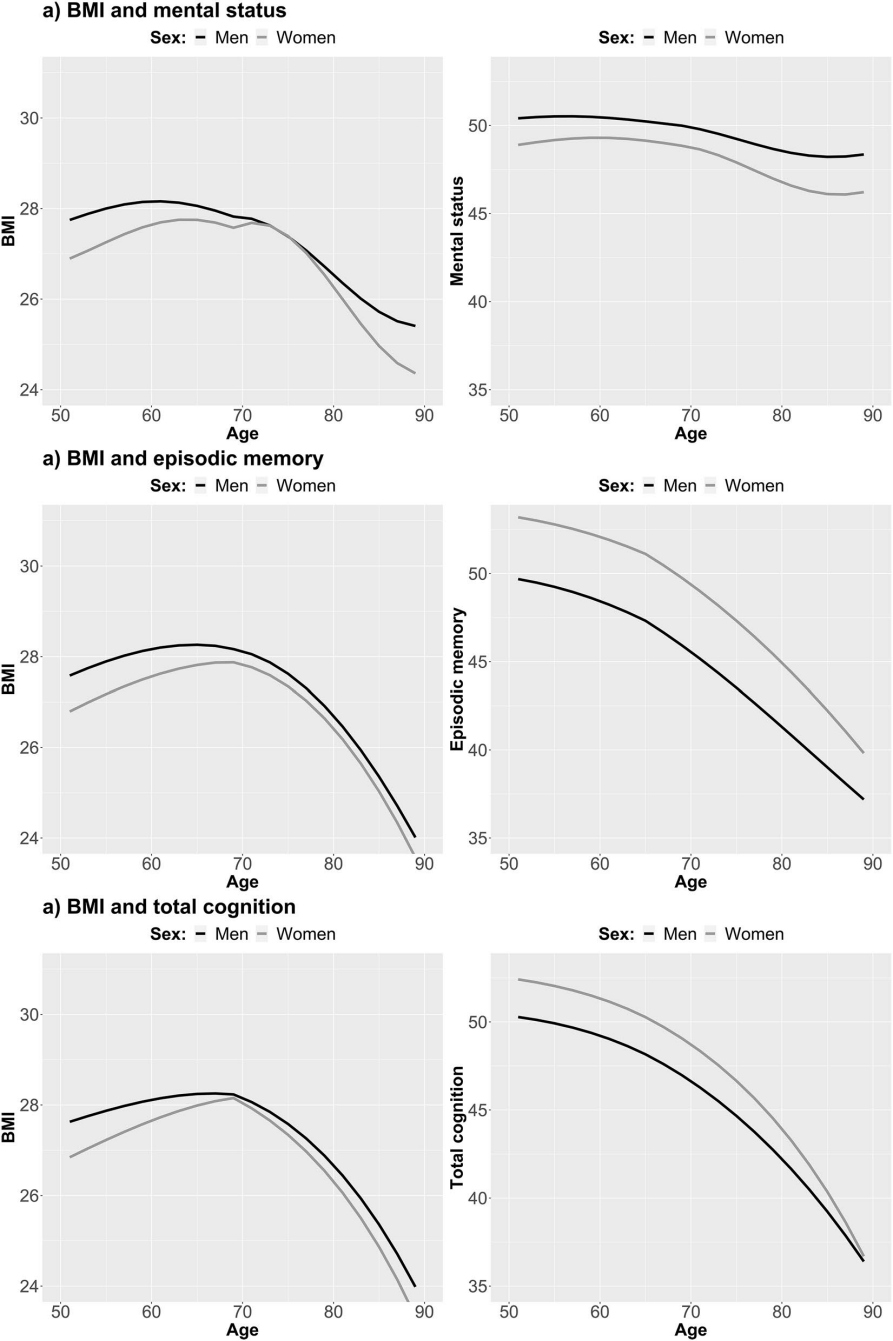
Longitudinal trajectories from bivariate dual change score models, showing change in BMI and cognitive abilities for men and women separately. Models were adjusted for education, and a breakpoint in the proportional change and coupling parameters added at age 70 for BMI and mental status, and at 65 for episodic memory and total cognition.

The sex-stratified change trajectories for BMI and cognitive abilities from the full coupling DCSM are shown in Fig. 4. The bivariate covariances between intercepts and slopes differed between men and women across BMI and all cognitive outcomes (p < 0.001 for all; Table S7), mostly such that covariances were stronger in women compared to men for mental status, but stronger in men compared to women for episodic memory and total cognition (Table S6a–c). In addition, the effect of BMI on change in episodic memory and total cognition (γ_BMI>COG_) was approximately twice or more as strong among men compared to women (p < 0.01 for both; Table S7; Table S6b,c). These BMI-driven effects, while smaller among women, were statistically significant before age 65 in both men and women, but not among women after age 65 (p = 0.17 and p = 0.34 for episodic memory and total cognition, respectively). No sex-differences were observed for the effect of BMI on change in mental status, or cognitive functioning on change in BMI (Table S7).

##### Differences by genetic predisposition to higher BMI

**Figure 5:**
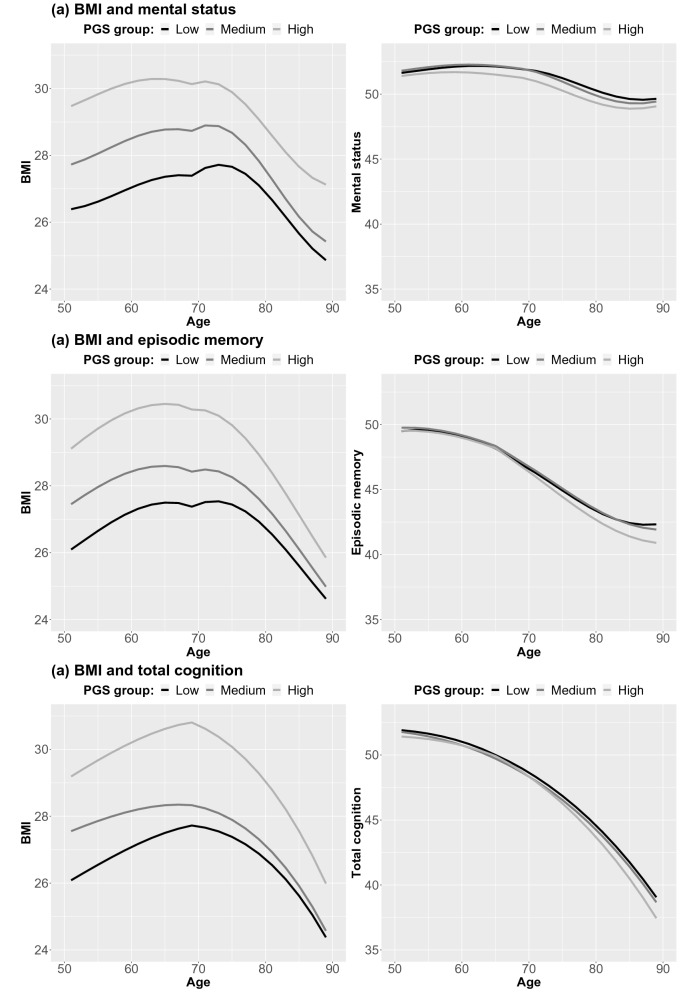
Longitudinal trajectories from bivariate dual change score models, showing change in BMI and cognitive abilities stratified by genetic predisposition to high, medium, or low BMI. All models were adjusted for sex and education, and a breakpoint in the proportional change and coupling parameters was added at age 70 for BMI and mental status, and at 65 for episodic memory and total cognition.

Change trajectories from the full coupling DCSM stratified by tertiles of the PGS_BMI_ are shown in Fig. 5. Bivariate covariances between intercepts and slopes differed by levels of the PGS_BMI_ for BMI and all the cognitive outcomes (p < 0.001 for all; Table S7), with generally stronger covariances observed with higher PGS_BMI_ (Table S6a–c). The effect of BMI on change in mental status and episodic memory (γ_BMI>COG_) also differed significantly (p = 0.02 and p < 0.001, respectively; Table S7), such that the effect of BMI on change in cognitive ability was generally stronger with lower PGS_BMI_ category (Table S6a,b). This is also seen comparing univariate trajectories (Figure S3) to bivariate trajectories (Fig. 5), where the largest differences are seen in the lower PRS categories. Notably, differences were rather small, and the parameter remained statistically significant across all PGS_BMI_ tertiles. The residual covariance between BMI and mental status and episodic memory also differed by PGS_BMI_ category (p < 0.01), with more residual covariance with higher PGS_BMI_.

#### Summary of results

For BMI, the univariate trajectory increased through age 70, after which it started to decrease. The pattern was more pronounced for women than for men, and the genetic risk had stronger influence on BMI at younger ages. In bivariate models, there was more decline in BMI when the overall effect of cognitive abilities was considered, but the effect was buffered by a higher cognitive ability, especially after age 70. This suggests that accelerating changes in BMI are influenced by cognition with maintenance of weight into late life a consequence of higher cognitive functioning. The effect was similar across sex and genetic propensity to high BMI.

For cognitive abilities, univariate trajectories generally declined already from age 50, but with a steeper decline after age 65 or 70. The pattern was more pronounced among women than men for episodic memory and total cognition. Baseline level of all cognitive domains were higher among those with a low PGS_BMI_. In bivariate models, the overall effect of BMI led to less decrease in cognitive abilities, but a higher BMI predicted steeper decline in cognitive abilities, starting from age 50. This suggests that accelerating changes in cognition are influenced by BMI with poorer maintenance of cognitive abilities being a consequence of higher BMI levels. Again, the effect was much stronger for mental status than for episodic memory and total cognition, where the effects were small though significant. The effect of BMI on change in episodic memory and total cognition were approximately twice as strong among men compared to women. In addition, the effect of BMI on change in mental status and episodic memory differed by genetic risk, with slightly stronger effects among those with genetic predisposition to a lower BMI.

## Discussion

In this study of the dynamic association between BMI and cognitive abilities, we used the powerful DCSM to evaluate the existence of a bidirectional relationship between BMI and cognitive abilities from age 50 through 89. While a higher cognitive ability was predictive of a more stable BMI, with less decline at later ages, a higher BMI predicted a steeper decline in cognitive abilities across midlife and late-life. Importantly, while the effect of BMI on change in cognitive ability was influenced by both sex and genetic predisposition to higher BMI, the effect of cognition on change in BMI was stable across groups. Taken together, our findings indicate that a higher BMI has a general negative effect on cognitive abilities throughout midlife and late-life, especially among men and in individuals with a genetically predicted low BMI. However, at the same time, individuals with declining cognition may experience a greater weight loss, and this effect is present regardless of sex and genetic propensity to high BMI.

The effect of BMI on cognition has been extensively studied (e.g.^36–40^). Taken together, the previous evidence indicates that midlife overweight and obesity is predictive of subsequent lower cognitive abilities, while late-life overweight and obesity appears to predict slower decline in cognitive abilities^41^. The same pattern has been seen in the effect of overweight on dementia, where midlife overweight is considered a well-established risk factor while late-life overweight may be associated with lower dementia risk^42^. This paradoxical protective effect of late-life overweight is hypothesized to stem from reverse causation, where unintentional weight loss is a sign of a preclinical dementia process^41^. Here, we demonstrated a negative effect of a high BMI on cognitive abilities that persist across old-age. However, we also demonstrated that higher cognitive ability predicted less decrease in BMI, especially at older ages. This finding strengthens the hypothesis of late-life weight loss being an indication of declining cognitive abilities, and the bidirectional association may explain the inconsistencies in previous studies of the effects of a higher BMI in late-life. It should be noted that the strongest effects were observed for the association between BMI and mental status. While the test of episodic memory will capture the small changes happening during normative cognitive aging, the mental status test will capture larger changes, potentially representing a preclinical dementia process^20^.

To the best of our knowledge only a few studies have considered the effect of cognition on change in BMI^1, 43–45^. The study by Hartanto and colleagues^1^ investigated the cross-lagged association between adiposity and cognitive function (executive functioning and episodic memory) at two assessments over 9 years (age 33–84 at baseline) and found a bidirectional association between waist-hip ratio, but not BMI, and cognitive abilities. Higher waist-hip ratio at baseline predicted lower episodic memory at follow-up in all age categories, and executive functioning among younger and middle-aged, but not older individuals. Conversely to our findings, where higher level of cognitive ability predicted less decline in BMI, they found that higher executive functioning, but not episodic memory, at baseline was predictive of lower waist-hip ratio at follow-up. As we here saw the strongest effect after age 70, the difference may be due to age differences in the samples. The difference in findings may also be explained by differences in cognitive measures and statistical model choice, as the study by Hartanto et al. studied rank order stability between 2 time points (over 9 years) while we focused specifically on the effect of cognitive functioning on change in BMI over the following 2 years, evaluating constant and dynamic growth processes.

We here identified stronger effects of a higher BMI on decline in episodic memory and total cognition among men than among women, and that the effect was only present among women before age 65. A recent study by Bohn et al.^9^ aimed to resolve inconsistencies in prior research on the subject by carefully studying sex-specific effects of BMI on longitudinal change in cognitive abilities (age 53–85 at baseline). In contrast to our findings, they found that higher BMI at baseline was associated with less decline in executive function, neurocognitive speed, and memory, but only among women. The direction of effect was thus in the opposite direction both of our findings and those from Hartanto. A potential explanation for these differences is that the study by Bohn and colleagues studied change over approximately 9 years in individuals with a mean age around 72 at baseline, and may be capturing the paradoxical effect of higher late-life BMI predicting better cognitive ability. Another explanation may be differences in cognitive measures. Indeed, in the current study covariances between BMI and cognition were stronger among women than men for mental status, while covariances and coupling effects between BMI and cognition were stronger in men for episodic memory and general cognition.

Our findings indicated small but significant genetic influences assessed by the PGS_BMI_, such that the effects of BMI on cognitive abilities were most prominent among those with genetic predisposition to a low BMI. Similarly, we have previously shown that a higher BMI in midlife predicts dementia only among those with a genetic predisposition to a low BMI^19^. This may indicate that a higher BMI due to environmental factors (such as a sedentary lifestyle and diet) has different consequences than a higher BMI due to genetic predisposition. However, our findings may also stem from the rather substantial negative genetic correlation between BMI and cognition, demonstrated to range from − 10 to − 51 using different methods^13^. This points to shared biological mechanisms influencing BMI and cognitive abilities, and potentially the association between them. Different biological hypotheses are reviewed by Farruggia and Small^2^, who conclude that the two most important factors likely are that obesity acts through chronic low-grade inflammation leading to neuronal damage or cardiovascular diseases, or that obesity may influence cognition through effects on cardiovascular fitness.

Strengths of this study include the large sample size with dense, longitudinal follow-up over many years, enabling us to study changes in both BMI and cognitive abilities from age 50 all the way through age 89. The HRS is a well-established, -documented, and -managed data source, with rich phenotypic information and additional genetic data. However, the study is not without limitations. First, the large sample size is not only a strength, but may lead to significant signals of very small effect sizes, not of practical or clinical relevance. Indeed, differences before and after age 70 as well as differences across groups were in some cases minimal, and we have aimed to highlight such issues and focus on meaningful effects. We were not able to include other measures of overweight, such as waist-hip ratio or waist circumference. Waist circumference is available from the face-to-face interviews in HRS, conducted regularly only from 2006 onwards (every 4 years) which would unfortunately not be enough time points to include in the DCSM setting. As we were mainly interested in longitudinal *change* in adiposity, BMI should capture the relevant information for the purpose. In addition, we only used self-reported BMI measures, but while this is not ideal and tends to result in lower BMI, these report biases are rather are stable over time^18^. It should also be noted that the index for mental status, and thus also total cognition, is only available at baseline and then from age 65. However, as mental status is generally expected to remain stable until older ages, no major changes would be expected at age 50–65 and this is consistent with what we found over this age range. We could only include individuals of European ancestry. The samples of other ancestries unfortunately had to sparse data, and since PGSs are ancestry specific the analyses would have had to be stratified accordingly. Studies of older adults introduce additional problems, with poor health being related to survival bias and attrition rate^46^. We acknowledge the risk that individuals experiencing a more substantial decline in BMI and/or cognitive abilities drop out to a larger extent, which may lead to an underestimation of the effects. However, as follow-up was repeated every 2 years and all measures were included, we could hopefully capture the individual trajectories prior to dropout also among those of poorer health.

In summary, we demonstrate a bidirectional relationship between BMI and cognitive abilities from age 50 through 89, where a higher BMI has a negative impact on cognitive functioning throughout midlife and late-life, while at the same time a higher cognitive functioning is protective against decline in weight, especially at older ages. The associations between BMI and mental status were rather strong, while those with episodic memory and total cognition were in the same direction, but substantially weaker. The effects of BMI on cognitive abilities were stronger among men and those with genetic predisposition to a lower BMI, while the effect of cognitive abilities on change in BMI was stable across sex and PGS_BMI_ groups. The two effects may thus stem from separate mechanisms, where overweight has a general negative effect on cognitive abilities throughout life, but at the same time weight-loss may be a sign of declining cognitive abilities.

## Supplementary Information

Supplementary Figures.

Supplementary Tables.

## Data Availability

The HRS and RAND files are public use datasets, available through: https://hrs.isr.umich.edu/data-products/access-to-public-data.

## Code availability

All codes used to generate analysis data and for conducting analyses are available upon request to the corresponding author.
