# Supplementary material for: The dynamic association between body mass index and cognition from midlife through late-life, and the effect of sex and genetic influences
Source: Sci Rep. 2021 Mar 30;11:7206. doi: 10.1038/s41598-021-86667-4 (PMC8010114; doi:10.1038/s41598-021-86667-4)
Supplement: Supplementary file 1 — Supplementary Figures. [file 41598_2021_86667_MOESM1_ESM.pdf]

The dynamic association between body mass index and cognition from midlife through late-life, and the effect of sex and genetic influences

Ida K. Karlsson*, Margaret Gatz, Thalida Em Arpawong, Anna K. Dahl Aslan, Chandra A. Reynolds

* ida.karlsson@ki.se

Supplementary figures 1-3

a) BMI

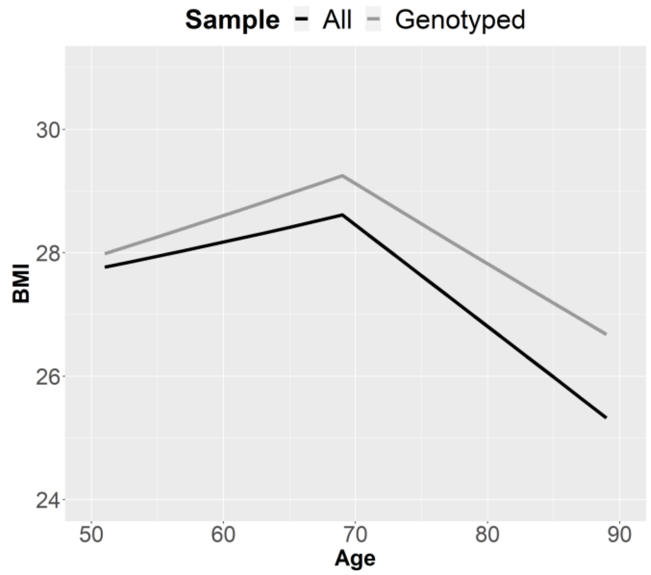

b) Mental status

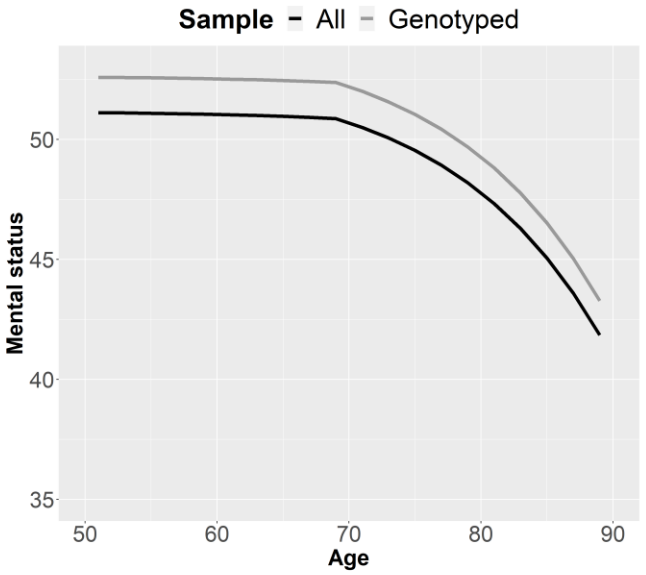

c) Episodic memory

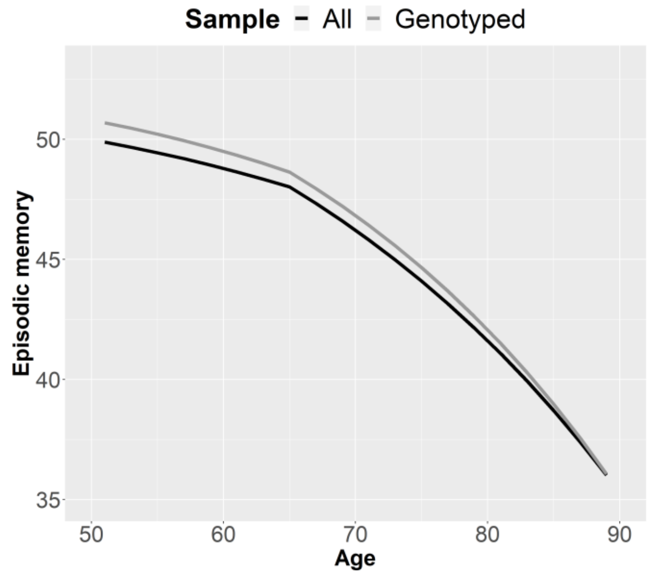

d) Total cognition

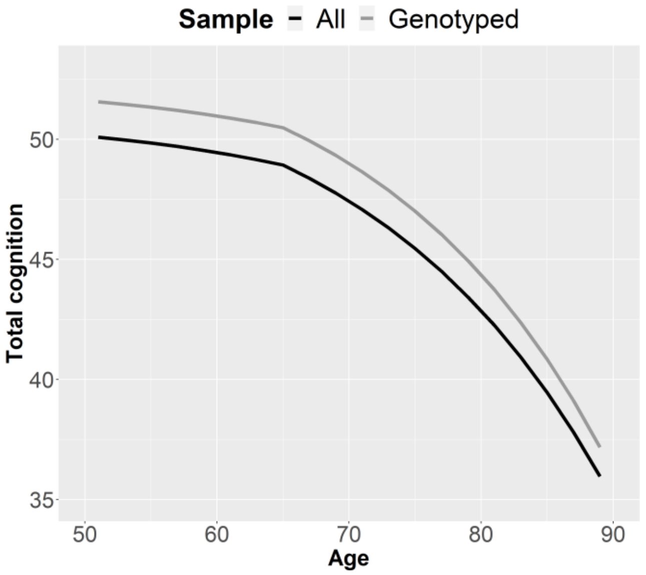

Figure S1: Longitudinal trajectories from univariate dual change score models, showing change in BMI and cognitive abilities in the full sample and in genotyped individuals. Trajectories are shown in black for the full sample and in grey for the genotyped sample. The dual change score models were adjusted for sex and education, and a breakpoint in the proportional change parameter added at age 70 for trajectories of BMI and mental status, and at age 65 for trajectories of episodic memory and total cognition.

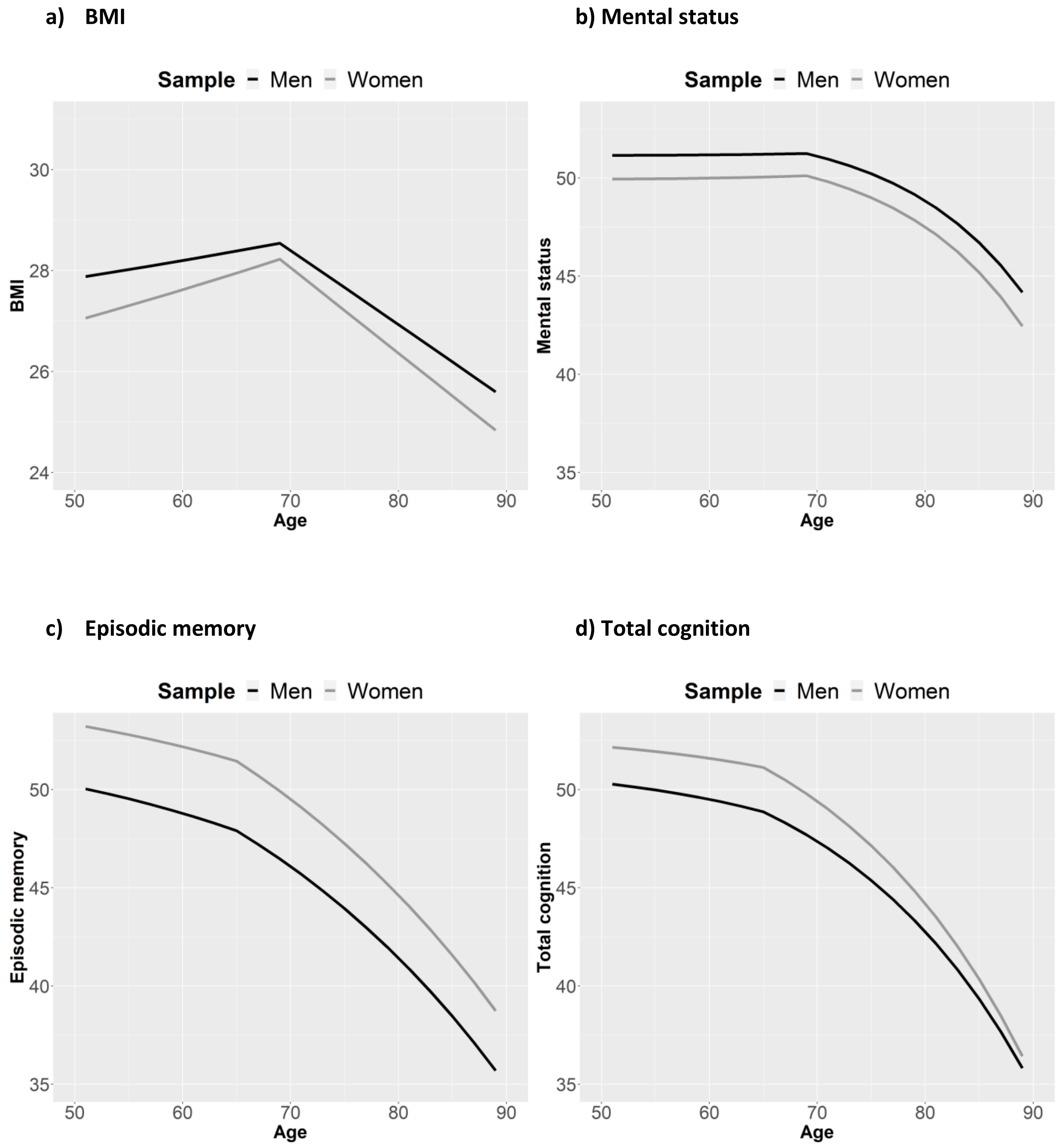

Figure S2: Longitudinal trajectories from univariate dual change score models, showing change in BMI and cognitive abilities in men and women separately. Trajectories are shown in black for men and in grey for women. The dual change score models were adjusted for sex and education, and a breakpoint in the proportional change parameter added at age 70 for trajectories of BMI and mental status, and at age 65 for trajectories of episodic memory and total cognition.

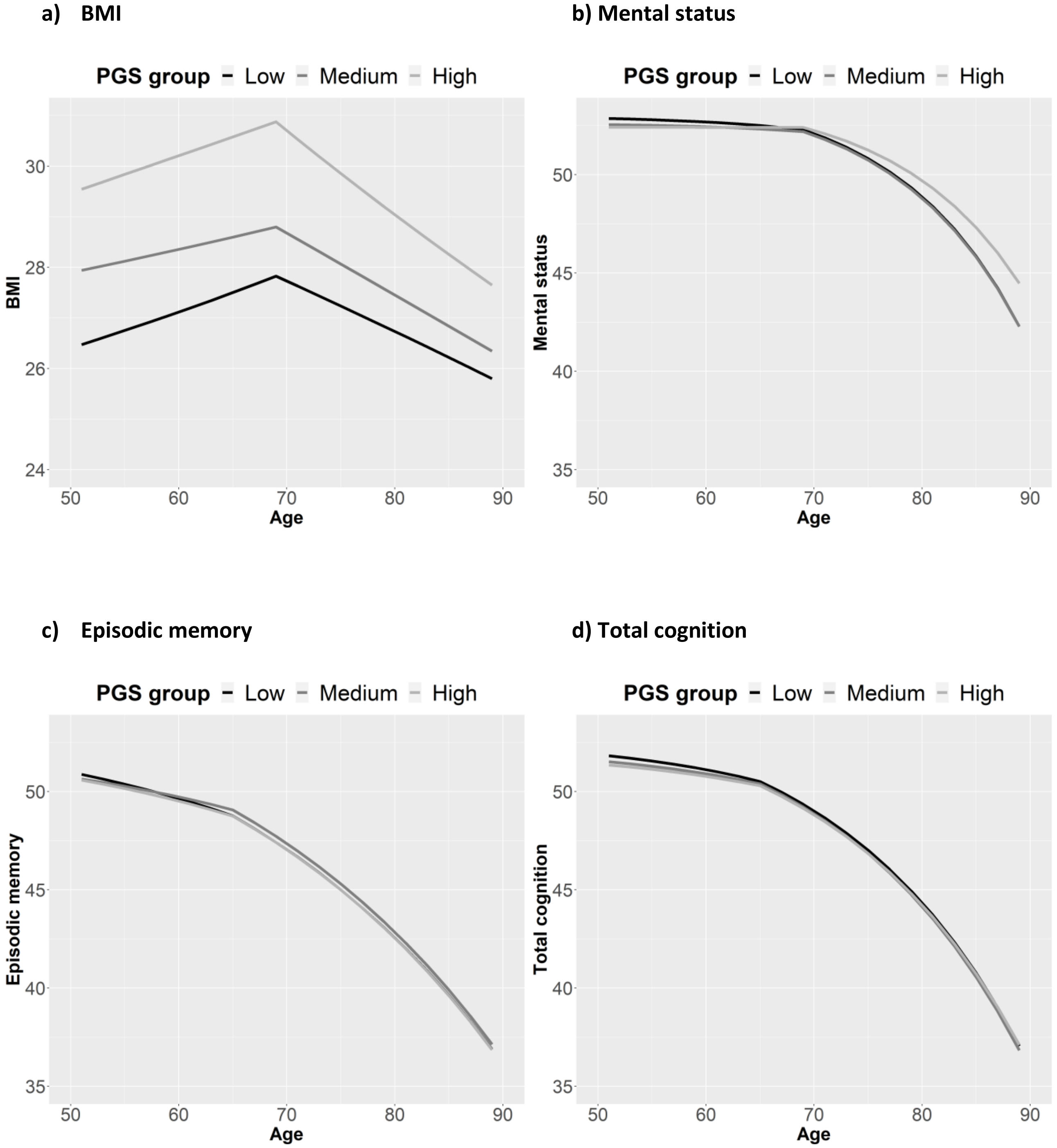

Figure S3: Longitudinal trajectories from univariate dual change score models, showing change in BMI and cognitive abilities stratified by genetic predisposition to low, medium, or high BMI. The trajectory for individuals in the lowest tertile of the polygenic score (PGS) for BMI are shown in black, those in the medium tertile in grey, and those in the highest tertile in light grey. The dual change score models were adjusted for sex and education, and a breakpoint in the proportional change parameter was added at age 70 for trajectories of BMI and mental status, and at age 65 for trajectories of episodic memory and total cognition.
